# Distinct cell proliferation, myogenic differentiation, and gene expression in skeletal muscle myoblasts of layer and broiler chickens

**DOI:** 10.1038/s41598-019-52946-4

**Published:** 2019-11-11

**Authors:** Yuma Nihashi, Koji Umezawa, Sayaka Shinji, Yu Hamaguchi, Hisato Kobayashi, Tomohiro Kono, Tamao Ono, Hiroshi Kagami, Tomohide Takaya

**Affiliations:** 1 Department of Agriculture, Graduate School of Science and Technology, Shinshu University, 8304 Minami-minowa, Kami-ina, Nagano 399-4598 Japan; 2 Department of Agricultural and Life Science, Faculty of Agriculture, Shinshu University, 8304 Minami-minowa, Kami-ina, Nagano 399-4598 Japan; 3 Department of Interdisciplinary Genome Sciences and Cell Metabolism, Institute for Biomedical Sciences, Shinshu University, 8304 Minami-minowa, Kami-ina, Nagano 399-4598 Japan; 4 NODAI Genome Research Center, Tokyo University of Agriculture, 1-1-1 Sakuragaoka, Setagaya-ku, Tokyo 156-8502 Japan; 5 Department of Bioscience, Tokyo University of Agriculture, 1-1-1 Sakuragaoka, Setagaya-ku, Tokyo 156-8502 Japan; 6 Present Address: Department of Embryology, Nara Medical University, 840 Shijo-cho, Kashihara, Nara 634-8521 Japan

**Keywords:** Transcriptomics, Muscle stem cells

## Abstract

Myoblasts play a central role during skeletal muscle formation and growth. Precise understanding of myoblast properties is thus indispensable for meat production. Herein, we report the cellular characteristics and gene expression profiles of primary-cultured myoblasts of layer and broiler chickens. Broiler myoblasts actively proliferated and promptly differentiated into myotubes compared to layer myoblasts, which corresponds well with the muscle phenotype of broilers. Transcriptomes of layer and broiler myoblasts during differentiation were quantified by RNA sequencing. Ontology analyses of the differentially expressed genes (DEGs) provided a series of extracellular proteins as putative markers for characterization of chicken myogenic cells. Another ontology analyses demonstrated that broiler myogenic cells are rich in cell cycle factors and muscle components. Independent of these semantic studies, principal component analysis (PCA) statistically defined two gene sets: one governing myogenic differentiation and the other segregating layers and broilers. Thirteen candidate genes were identified with a combined study of the DEGs and PCA that potentially contribute to proliferation or differentiation of chicken myoblasts. We experimentally proved that one of the candidates, enkephalin, an opioid peptide, suppresses myoblast growth. Our results present a new perspective that the opioids present in feeds may influence muscle development of domestic animals.

## Introduction

In the past half-century, genetic selection has improved growth rate of broiler chickens, to cater to the increased demand of chicken meat worldwide^1^. As a result, commercial broiler chickens have been engineered to quickly develop compared to other breeds such as layer chickens^2,3^. Development and growth of broiler and layer chickens are markedly different. Body weights at hatching are 44.4 g in broilers but 38.5 g in layers^3^. Post-hatch growth ratio of broilers is several times higher than that of layers. Body weights of broilers reach over 2 kg within 4–5 weeks but those of layers are still 0.4 kg^2,3^. For further advancement of selective breeding, feed efficiency, and meat yield, numerous studies have been performed to understand the molecular mechanisms underlying the phenotypes of broiler chicken. Quantitative trait loci (QTL) mapping analysis of F_2_ chickens generated by crossing broiler and layer chickens indicated that a limited number of chromosomal regions appear to govern body weight^4^. Microarray study using duodenum samples reported that oxidative stress, DNA damage, and apoptosis are influenced by feed intake^5^.

The difference of body weights between layers and broilers is mainly due to the size of skeletal muscle. Broiler muscles have a greater number of the myofibers showing larger diameters compared to that in layers^2^. Skeletal muscle tissue as meat has been a critically important target for comprehensive gene expression analysis in broiler chickens. Microarray analyses of the breast muscles revealed that the genes related to slow-type myofibers, satellite cell proliferation, and muscle hypertrophy are differentially expressed between layers and broilers^6^. Even in a single genetic line of male broilers, gene expression patterns in breast muscles differ associated with feed efficiency^7,8^.

More recently, RNA sequencing (RNA-seq) has become a powerful method for precise quantification of not only coding but also non-coding RNAs. With recent improvements of chicken genome information, RNA-seq successfully revealed the intriguing genes affecting egg albumen in layers^9^ and comb size of cocks^10^. The latest studies using RNA-seq have also reported differential gene expression profiles in breast muscles of modern and foundation broiler lines^11,12^ or those in broiler lines with high- and low-ultimate pH^13^. These data provided a list of candidate genes that may be involved in muscle development, stress response, or meat quality in chickens. However, muscle tissue is comprised of not only myogenic cells but also a variety of cell types including adipocytes, fibrocytes, and vascular cells. In addition, muscle growth after hatching can be affected by external factors such as feed, temperature, and various environmental conditions. For more strict comparison of myogenic cells among chicken breeds, a well-regulated experimental system without the influence of extrinsic factors is required.

Myofibers are multi-nucleated giant myocytes that are the main components of skeletal muscle tissue. Each myofiber has a number of somatic stem cells, termed satellite cells, located between the basal lamina and cell membrane of myofibers. During muscle development and growth, quiescent satellite cells are activated to proliferative myoblasts as myogenic progenitor cells. After several rounds of cell division, myoblasts arrest cell cycle and terminally differentiate into mononuclear contractile myocytes. Finally, myocytes fuse to existing myofibers or fuse each other in order to form multinuclear myotubes. These nascent myotubes mature into fully functional myofibers with organizing actomyosin filaments to acquire adequate contractile ability^14^. Therefore, myoblasts play a central role during muscle formation, and partly reflect the muscle phenotype of animals. However, to date, there have been no data on inclusive quantification of the genes expressed in chicken myoblasts.

In the present study, we investigated the myoblasts isolated from layer and broiler chicken embryos as congenital cell sources of skeletal muscle. Since proliferative and differentiative abilities of the myoblasts satisfactorily corresponded to the muscle phenotypes, we analyzed global gene expression profiles of their myogenic cells during differentiation by RNA-seq, and subsequently tried to identify candidate genes that potentially contribute to the properties of chicken myoblasts.

## Results

### Proliferative and differentiative characteristics of layer and broiler chicken myoblasts

**Figure 1:**
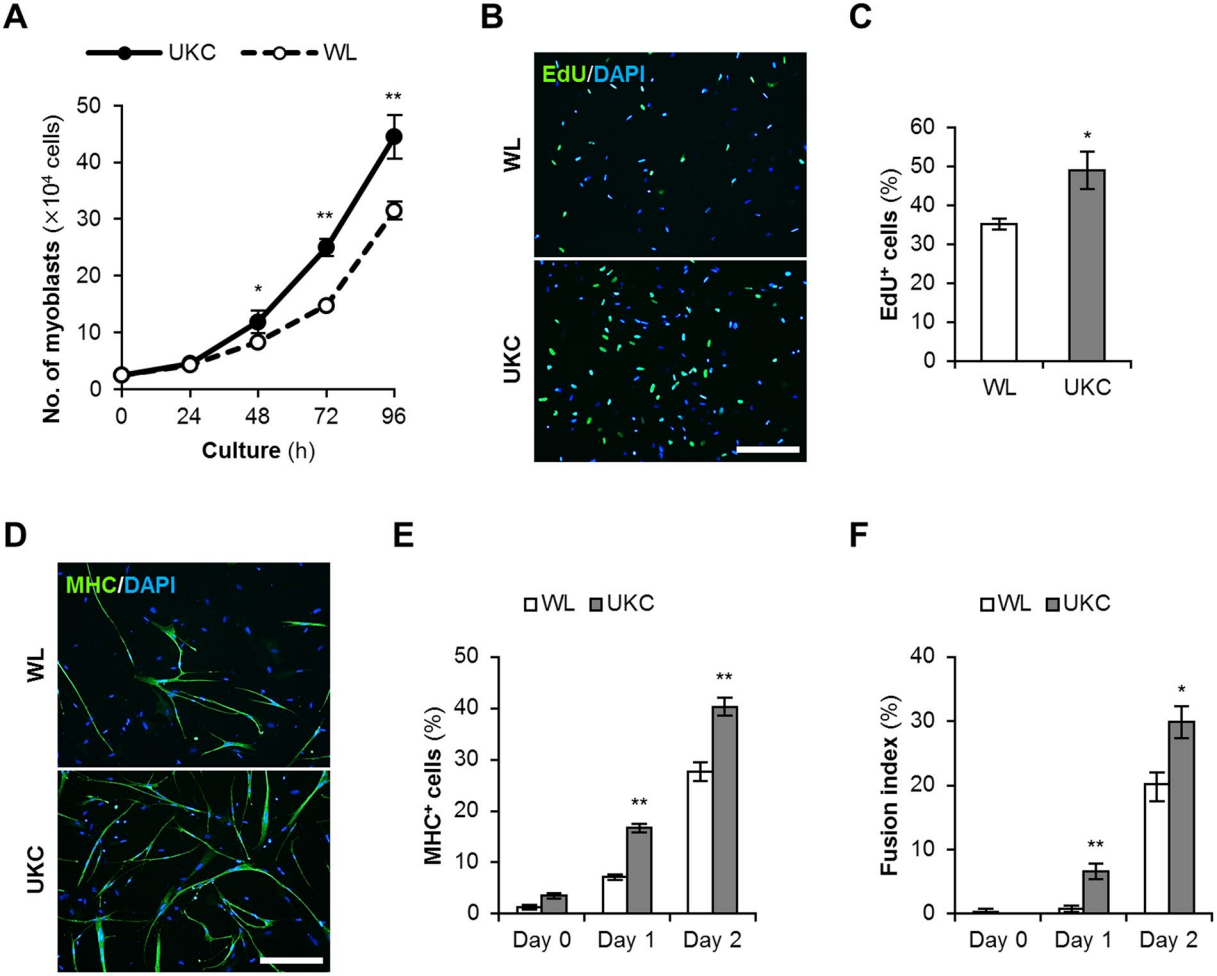
Proliferation and differentiation of chicken myoblasts. **(A)** Growth of chicken myoblasts in GM. *p < 0.05; **p < 0.01 (Student’s *t* test at each time point). *n* = 3. **(B)** EdU staini*n*g of chicken myoblasts in GM. Scale bar, 200 μm. **(C)** The ratio of EdU^+^ cells. *p < 0.05 (Student’s *t* test). *n* = 4. **(D)** MHC staining of chicke*n* myoblasts in DM at day 2. Scale bar, 200 μm. **(E**,**F)** The ratio of MHC^+^ myocytes (**E**) and fusion indexes (**F**). *p < 0.05; **p < 0.01 vs WL at each time point (Student’s *t* test). *n* = 4.

Chicken myoblasts were isolated from hindlimbs of White Leghorn (WL) layer and UK Chunky (UKC) broiler embryos at Hamburger-Hamilton stage (HH) 36. Proliferation of these myoblasts were examined by counting the cell numbers every 24 h. From 48 h through 96 h after seeding, the numbers of UKC myoblasts were significantly higher than those of WL myoblasts (Fig. 1A). Their proliferative abilities were quantified by measuring DNA synthesis *via* the incorporation of 5-ethynyl-2′-deoxyuridine (EdU) (Fig. 1B). The rate of EdU^+^ UKC myoblasts (49.0 ± 4.8%) was significantly higher than that of WL myoblasts (35.1 ± 1.4%) (*p* = 0.032) (Fig. 1C). These data indicate that UKC myoblasts proliferate faster and generate more progenies than WL myoblasts.

Next, WL and UKC myoblasts were induced myogenic differentiation then immunostained for myosin heavy chain (MHC), a terminal differentiation marker of skeletal muscle cells (Fig. 1D). The ratio of MHC^+^ myocytes and multinuclear myotubes were significantly higher for UKC myoblasts than for WL myoblasts on day 1 and day 2 of differentiation (Fig. 1E,F), demonstrating that UKC myoblasts differentiate into MHC^+^ myocytes and form mature myotubes more rapidly than WL myoblasts.

The characteristics of UKC myoblasts, which are more proliferative and differentiative than WL myoblasts, corroborated with the muscle phenotypes of broiler chickens. Our data suggest that the genes expressed in UKC myoblasts might be involved in the robust muscle development and growth of broiler chickens.

### Differentially expressed genes between layer and broiler chicken myogenic cells

For comprehensive gene expression analyses, WL and UKC myoblasts were induced myogenic differentiation three times, independently. Total RNA of the cells on days 0, 1, and 2 of myoblast differentiation was isolated and subjected to RNA-seq. After carrying out quality control on the raw reads, 23.3 million reads per sample were acquired, of which approximately 22.6 million reads (97.2%) were mapped to the chicken reference genome (Gallus_gallus-5.0) (Supplementary Table S1). In total 26,640 genes were identified from 46,389 transcripts. Gene expression levels were calculated as the reads per kilobase per million reads (RPKM).

**Figure 2:**
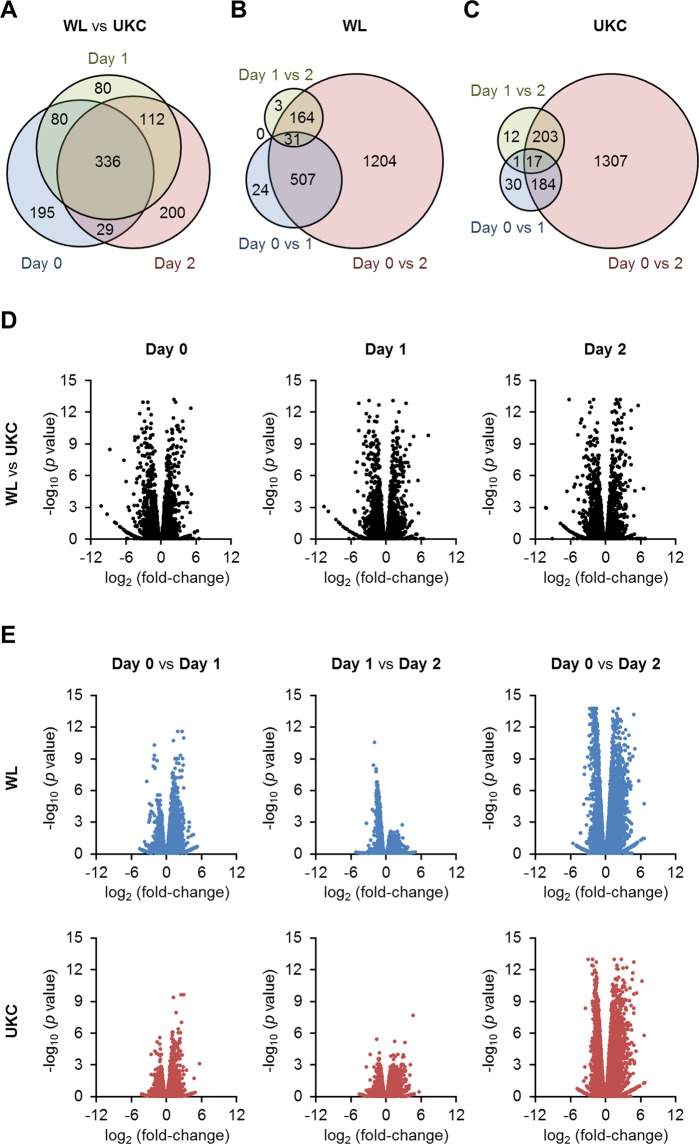
DEGs in chicken myoblasts. **(A)** Numbers of DEGs between WL and UKC myoblasts on each day. **(B,C)** Numbers of DEGs during differentiation of WL (**B**) and UKC (**C**) myoblasts. *p* < 0.05 (FDR), |fold-change| ≥ 2. **(D**,**E)** Volcano plots of the DEGs shown in (**A**–**C**).

The differentially expressed genes (DEGs) among the samples were defined by considering *p* value of false discovery rate (FDR) < 0.05 and |fold-change| ≥ 2 as cutoffs. First, the DEGs were screened by comparing WL and UKC myogenic cells on each day of differentiation. As shown in Fig. 2A,D, a total of 1,032 DEGs were identified, of which 336 DEGs (171 upregulated and 165 downregulated in UKC) were differentially expressed throughout myogenic differentiation from day 0 to day 2 (Supplementary Data 1). These 336 DEGs were considered to underlie the differences in cellular characteristics of WL and UKC myogenic cells. Gene ontology (GO) analysis revealed that the 336 DEGs significantly form some functional gene clusters (Table 1). Notably, the 336 DEGs were enriched for extracellular and cell surface proteins such as collagens, channels, receptors, and ligands. These proteins possibly reflect the characteristics of myogenic cells and may be useful as cell markers to predict muscle development of chicken breeds.

**Table 1:** Gene ontologies of the 336 DEGs.

Class	*p*	Genes
Proteinaceous extracellular matrix	7.1E-06	*NOV*, *HAPLN1*, *COL9A2*, *COL14A1*, *CRISPLD2*, *SMOC1*, *LUM*, *ELN*, *COL6A3*, *FBN3*, *ECM2*, *COL16A1*, *SLIT3*
Extracellular matrix	3.4E-05	*COL14A1*, *COL21A1*, *COMP*, *F3*, *MMP27*, *MMP17*, *FBN3*, *TGM4*, *AGRN*, *FBN2*
Extracellular region	7.7E-05	*FGF19*, *BGLAP*, *CCK*, *ENPP2*, *C1R*, *ESM1*, *PTGFR*, *ADCYAP1*, *NOV*, *PGR*, *DNASE1*, *DKK3*, *BMPER*, *CXCL14*, *AGRN*, *NRG1*, *CD200R1*
Extracellular space	3.6E-04	*ENPP2*, *PDGFA*, *LUM*, *FAM132A*, *LRRC4C*, *NRN1*, *GREM2*, *ABI3BP*, *C1QTNF5*, *COMP*, *COL6A3*, *SERPINB10*, *PTN*, *NRG1*, *LOC422654*, *ECM2*, *SLIT3*, *PTHLH*, *GKN2*, *BMPER*, *COL14A1*, *F3*, *SEMA4D*, *IL12B*, *LIPC*
Axon	2.7E-03	*ALCAM*, *PTPRK*, *CCK*, *PVALB*, *STMN2*, *CHRNA7*, *AGRN*, *GAP43*
Neuron projection	3.4E-03	*STMN2*, *TENM2*, *MAP2*, *AGRN*, *GHSR*, *GAP43*, *TPH2*, *ADCYAP1*
Integral component of plasma membrane	1.0E-02	*SLC8A3*, *LPPR1*, *FLT4*, *TRPV3*, *PTGFR*, *APCDD1*, *KDR*, *ALCAM*, *SEMA6A*, *THBD*, *SLC24A4*, *SLC6A7*, *SLC24A2*, *TENM2*, *ADRA1B*, *CLDN1*, *TBXA2R*, *SEMA4D*, *NRG1*, *FAM26F*
Collagen trimer	2.7E-02	*COL9A2*, *C1QTNF5*, *COL14A1*, *COL6A3*
Cell surface	3.9E-02	*PTPRK*, *THBD*, *CAPN5*, *CLSTN2*, *PDGFA*, *F3*, *BF2*, *ITGB5*, *GHSR*, *MDK*

### DEGs during chicken myogenic differentiation

In addition to the 336 DEGs, other DEGs whose expression was significantly changed during myogenic differentiation were listed for the two types of myoblasts. As shown in Fig. 2B,C, a total of 1,754 and 1,933 DEGs were identified in WL and UKC myogenic cells, respectively. Volcano plots showed that the shifts in gene expression within 24 h (from day 0 to day 1 or from day 1 to day 2) were not drastic in both the types of myogenic cells (Fig. 2E). The major populations of the DEGs (68.6% in WL and 67.6% in UKC) required 48 h (from day 0 to day 2) to show alteration in their expression levels.

**Figure 3:**
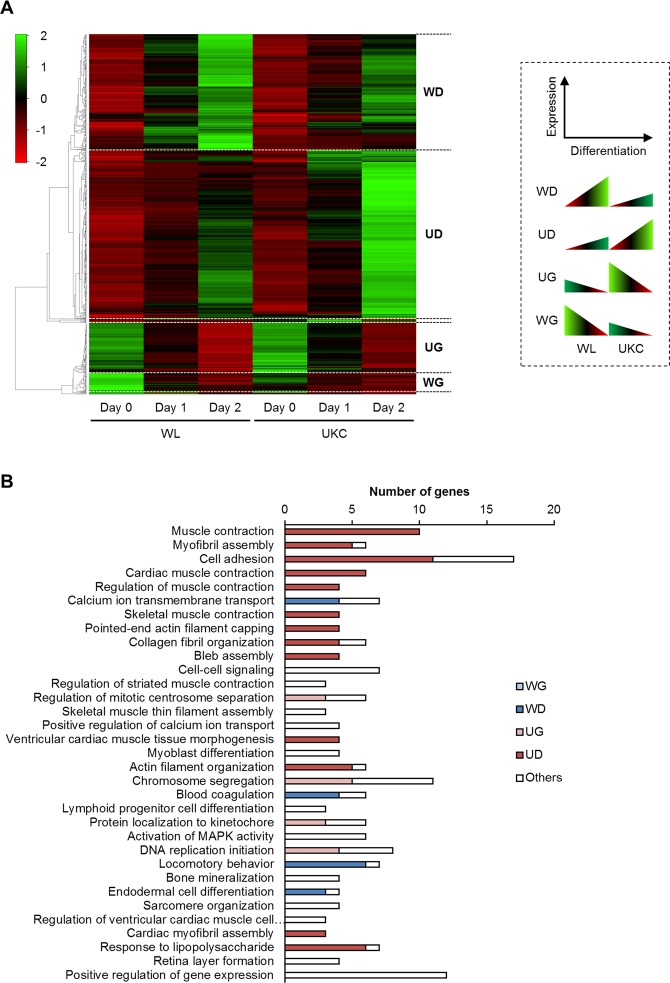
DEGs involved in myogenic differentiation. **(A)** Heatmap and phylogeny of the 840 DEGs during differentiation. *p* < 0.05 (FDR), |fold-change| ≥ 4. **(B)** Ontology analysis of the 840 DEGs.

**Figure 4:**
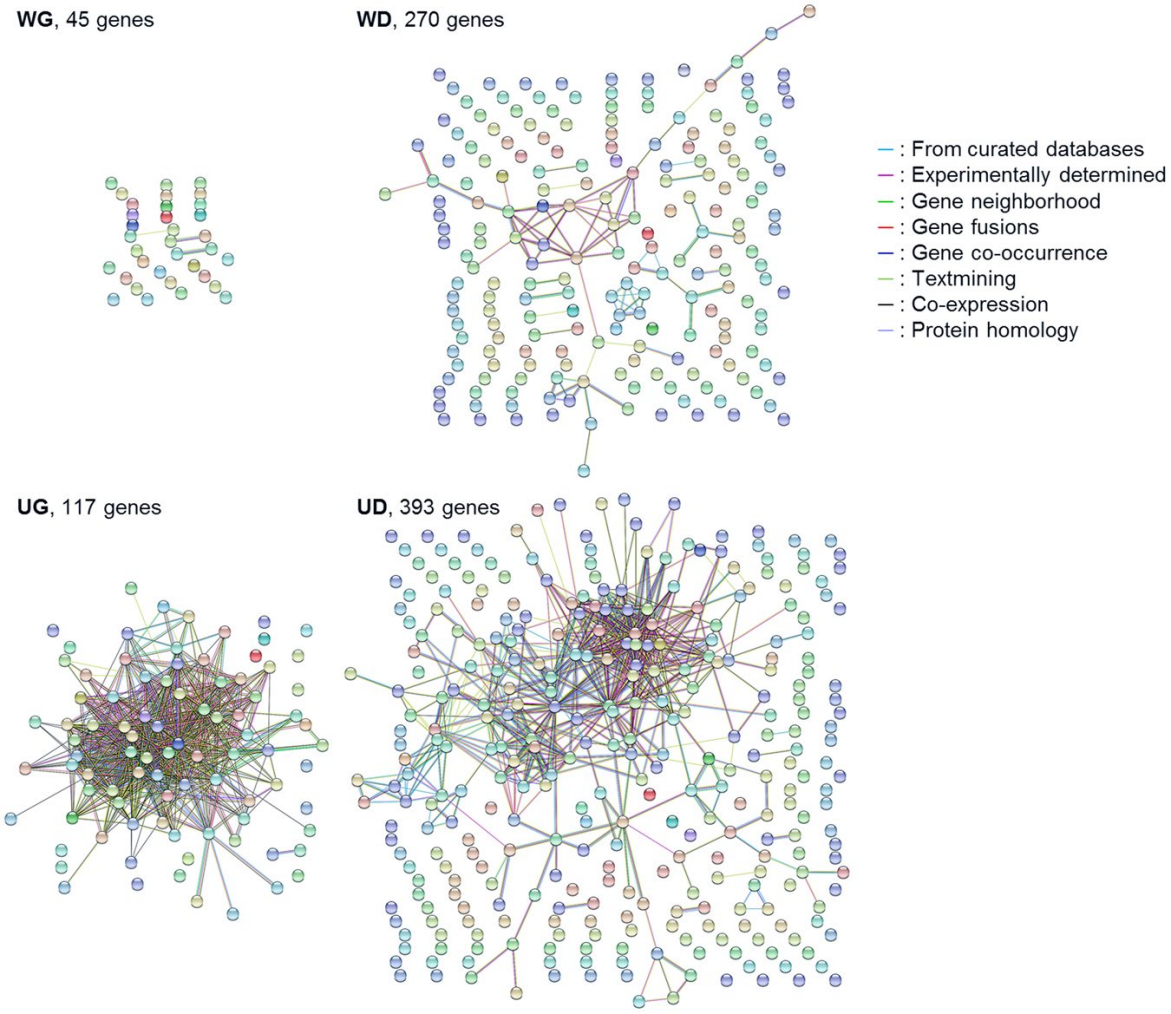
Functional protein association networks within WG, WD, UG, and UD genes were visualized by STRING.

To investigate the functions of the genes involved in chicken myogenic differentiation, a novel set of the DEGs was defined by considering *p* value of FDR < 0.05 and |fold-change| ≥ 4 during differentiation (day 0 vs day 1, day 1 vs day 2, or day 0 vs day 2) as cutoffs in WL and UKC myogenic cells. These thresholds defined the 840 DEGs with altered transcription levels as some point in myogenic differentiation in either of the breeds (Supplementary Data 2). The heatmap for the 840 DEGs clearly showed the genes that were upregulated or downregulated during differentiation of WL and UKC myogenic cells (Fig. 3A). Hierarchical clustering classified the 840 DEGs into four subgroups: WG (WL growth), WD (WL differentiation), UG (UKC growth), and UD (UKC differentiation). 45 WG genes were highly expressed in the growing WL myoblasts at day 0, and 270 WD genes were significantly induced in the differentiated WL myogenic cells at day 2. Similarly, 117 UG genes and 393 UD genes were highly transcribed in the proliferating and differentiated UKC myogenic cells, respectively. GO analysis indicated that the 840 DEGs significantly formed multiple gene clusters for cell cycle regulation and muscle formation (Fig. 3B). Some cell cycle-related clusters (for example, regulation of mitotic centrosome separation, chromosome segregation, and DNA replication initiation) had abundant UG genes, and many muscle clusters (for example, muscle contraction, myofibril assembly, and actin filament organization) were rich in UD genes. These distributions of the DEGs corresponded well with the characteristics of UKC myoblasts that show active proliferation and differentiation. Interactions of the genes or their products in each subgroup were visualized using the STRING database (Fig. 4). The data suggest that the genes within UG/UD tightly interact with each other, but those within WG/WD do not. GO analyses of the subgroups also showed that UG and UD were significantly related to the gene clusters for cell cycle regulation and muscle formation, respectively (Supplementary Table S2). However, such clusters were not detected in WG and WD. These data illustrate that the potent proliferative and differentiative abilities of UKC myoblasts are based on the expression of UG and UD genes.

### Identification of candidate genes by principal component analysis

**Figure 5:**
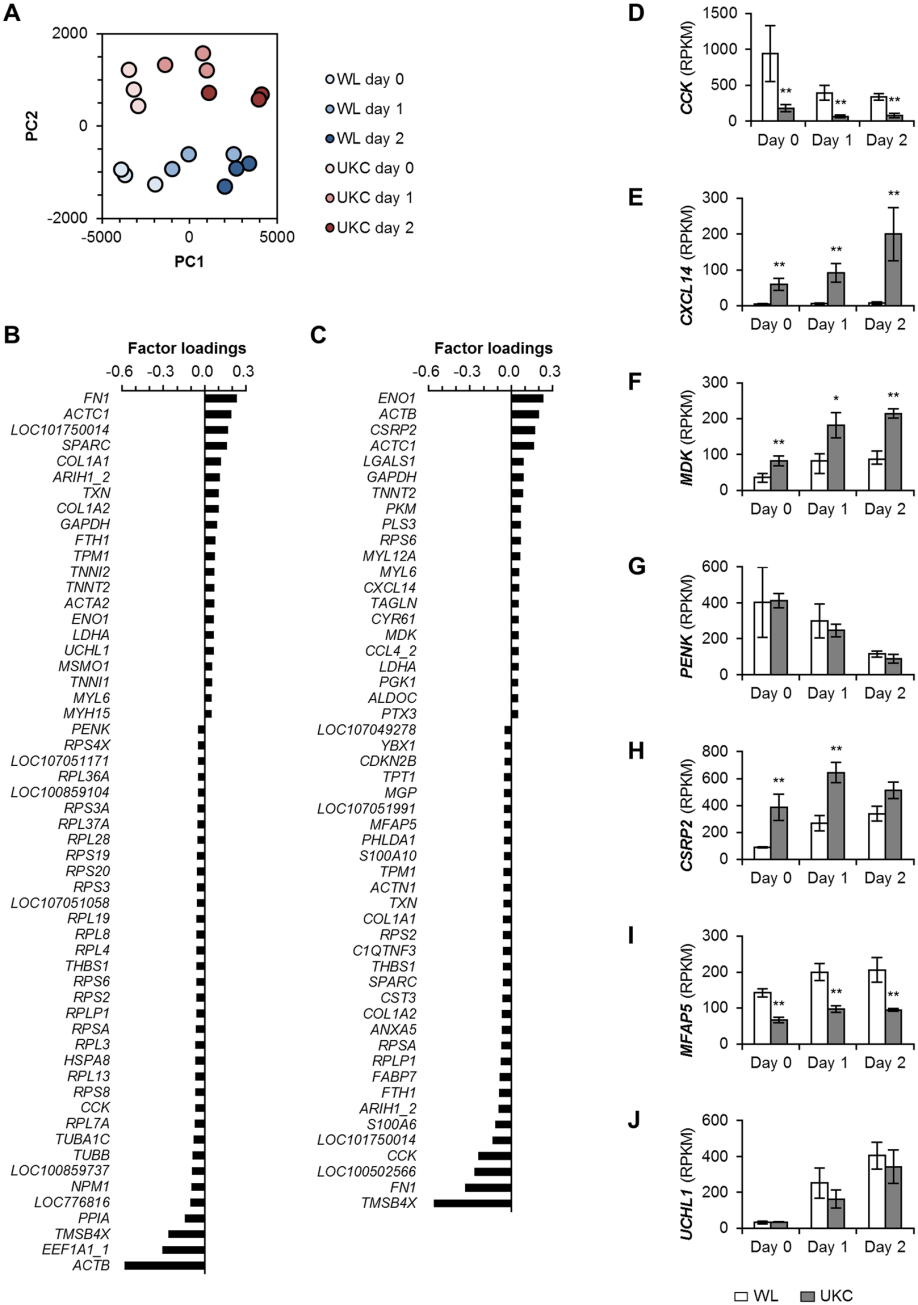
Results of PCA and expression levels of the candidate genes. **(A)** PCA space with PC1 and PC2. Total 18 samples as presented in the legend were plotted. **(B**,**C)** Genes with significant factor loadings for PC1 (**B**) and PC2 (**C**) are listed in a descending order. The significant value of factor loadings is selected by criterion that the value is larger than μ + 5σ or smaller than μ-5σ. μ, mean value; σ, standard deviation. **(D**–**J)** RPKM values of RNA-seq as transcription levels of *CCK* (D), *CXCL14* (**E**), *MDK* (**F**), *PENK* (**G**) *CSRP2* (**H**), *MFAP5* (**I**), and *UCHL1* (**J**) genes. *p < 0.05; **p < 0.01 vs WL at each time point (FDR).

Semantic GO analysis is a powerful method to understand the major properties of a gene set but is unable to ascertain the importance of unknown genes. To explore the factors determining properties of chicken myogenic cells, principal component analysis (PCA) was performed using the RPKM values of all the 18 samples. PCA worked out the first and second principal components (PC1 and PC2) with contributions 0.73 and 0.10, respectively. Subsequently, the 18 samples were plotted on the PCA space reconstructed by PC1 and PC2 (Fig. 5A). The PC1 axis satisfactorily corresponded to the differentiation stages of both WL and UKC myogenic cells. Thus, we assumed that the genes with significant factor loadings for PC1 (Fig. 5B) are involved in myogenic differentiation. Furthermore, the PC2 axis clearly revealed dissimilarities between WL and UKC myogenic cells, indicating that the genes with significant loadings for PC2 (Fig. 5C) may contribute to the breed-dependent differences.

To refine the search for candidate genes with important functions in chicken myogenic cells, the PCA-identified genes included in the 336 and 840 DEGs were screened. As a result, 13 candidate genes were identified (Table 2). As expected, the 13 candidates included well-known genes in myogenic cells: one cell cycle-related factor (*CDKN2B*) and five sarcomeric components (*ACTC1*, *MYH15*, *TNNI1*, *TNNI2*, and *TNNT2*). These results support the fact that these candidates successfully reflect the key functions of chicken myogenic cells. In addition to these well-defined genes, the candidates also contained other seven genes (*CCK*, *CXCL14*, *MDK*, *PENK*, *CSRP2*, *MFAP5*, and *UCHL1*) which showed discriminatory expression patters during myogenic differentiation (Fig. 5D–J). Since their precise functions in myogenic cells have been still unclear not only in aves but also in mammals, the roles of these candidates needed to be experimentally investigated. In the following study, we focused on *PENK* gene encoding pro-enkephalin peptide. Pro-enkephalin derives two forms of pentapeptides, methionine-enkephalin (MENK) and leucine-enkephalin (LENK), which are the endogenous opioid peptides involved in regulating nociception^15,16^. However, their actions on myoblasts have not been reported. Moreover, enkephalin-like exogenous opioid peptides are known to present in various feed^17^. Therefore, understanding of the effects of enkephalin on myoblasts is considered to contribute to improve not only muscle development but also feed efficiency.

**Table 2:** The 13 candidate genes.

Category	Gene	Product	Involved in	
Cell cycle	*CDKN2B*	Cyclin-dependent kinase inhibitor 2B (p15)	PC2	336 DEGs
Muscle	*ACTC1*	Cardiac muscle actin, alpha	PC1, PC2	840 DEGs
	*MYH15*	Myosin heavy chain 15	PC1,	840 DEGs
	*TNNI1*	Troponin I1	PC1	840 DEGs
	*TNNI2*	Troponin I2	PC1	840 DEGs
	*TNNT2*	Troponin T2	PC1, PC2	840 DEGs
Peptide, cytokine	*CCK*	Cholecystokinin	PC1, PC2	336 DEGs
	*CXCL14*	C-X-C motif chemokine ligand 14	PC2	336 DEGs
	*MDK*	Midkine	PC2	336 DEGs
	*PENK*	Proenkephalin	PC1	840 DEGs
Others	*CSRP2*	Cysteine and glycine-rich protein 2	PC2	840 DEGs
	*MFAP5*	Microfibril associated protein 5	PC2	336 DEGs
	*UCHL1*	Ubiquitin C-terminal hydrolase L1	PC1	840 DEGs

### Enkephalins suppress chicken myoblast proliferation

**Figure 6:**
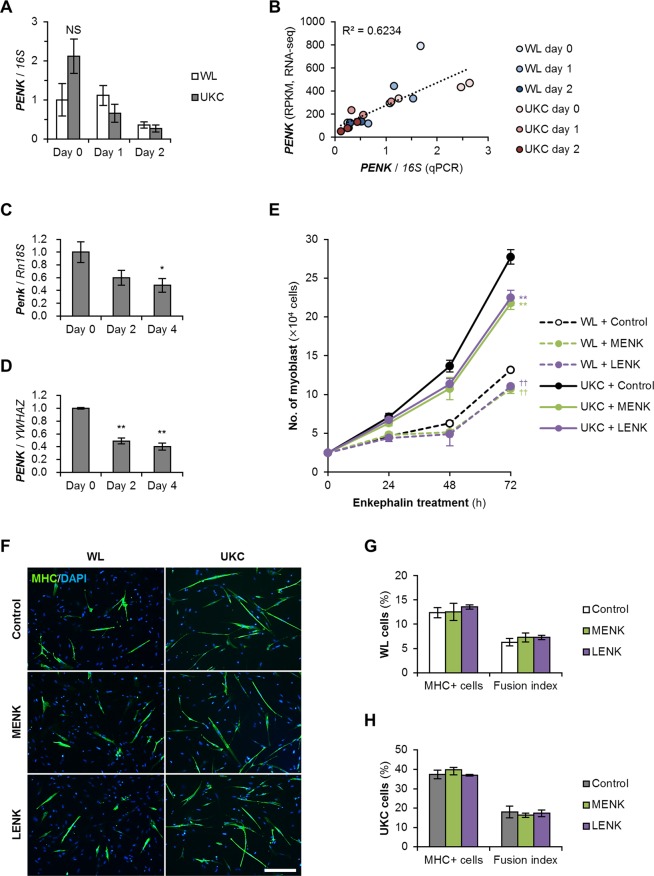
Enkephalin suppresses myoblast proliferation. **(A)** qPCR results of *PENK* gene transcription in the chicken myoblasts subjected to RNA-seq. NS, no significant difference vs WL (Student’s *t* test at each time point). *n* = 3. **(B)** Correlation of the chicken *PENK* gene transcription quantified by RNA-seq and qPCR (Pearson’s correlation coefficient test). **(C**,**D)** qPCR results of pro-enkephalin gene transcription in murine (**C**) and human (**D**) myoblasts in DM. The mean value at day 0 was set at 1.0. * *p* < 0.05, **p < 0.01 vs day 0 (Williams’ test). *n* = 3–4. **(E)** Growth of WL and UKC myoblasts in GM with 1 μM MENK or LENK peptide. ^††^*p* < 0.01 vs control WL; **p < 0.01 vs control UKC (Scheffe’s *F* test at each time point). *n* = 4. **(F)** MHC staining of WL and UKC myoge*n*ic cells in DM with 1 μM enkephalin at day 2. Scale bar, 200 μm. **(G**,**H)** The ratio of MHC^+^ myocytes and fusion indexes of WL (**G**) and UKC (**H**) myogenic cells. No significant difference among control, MENK, and LENK samples in each group (Scheffe’s *F* test). *n* = 4.

Expression levels of chicken *PENK* gene were re-confirmed by quantitative real-time RT-PCR (qPCR) (Fig. 6A). The values obtained by RNA-seq and qPCR were strongly correlated (*R*^2^ = 0.623) (Fig. 6B), indicating that chicken *PENK* gene expression is downregulated during myotube formation but is not significantly different between WL and UKC myogenic cells. Although a previous study showed that rat L6 myoblast cell line transcribe *Penk* mRNA^18^, it has not been reported whether mammalian primary-cultured myoblasts actually express pro-enkephalin genes through myogenic differentiation as in chickens. We confirmed the differentiation-dependent declines of murine *Penk* and human *PENK* gene expression in primary-cultured juvenile murine and adult human myoblasts (Fig. 6C,D), demonstrating that avian and mammalian myoblasts produce enkephalin in common.

To ascertain the effects of enkephalins on the growth of chicken myoblasts, the numbers of the WL and UKC myoblasts treated with 1 µM of MENK or LENK peptide were counted every 24 h. Both MENK and LENK significantly decreased the numbers of WL and UKC myoblasts at 72 h after the treatment (Fig. 6E). In contrast, neither MENK nor LENK peptide affected differentiation of WL and UKC myoblasts into MHC^+^ myotubes (Fig. 6F–H). These data indicate that enkephalins are able to suppress proliferation but not affect differentiation of chicken myoblasts.

## Discussion

The present study demonstrated marked differences in myoblasts between layer WL and broiler UKC. Compared to WL myoblasts, UKC myoblasts proliferated faster in growth condition, and subsequently, differentiated more rapidly into MHC^+^ myotubes *in vitro*. These characteristics of UKC myoblasts corresponded well with the phenotype of the broilers that produce well-developed muscle tissues with a large number of myofibers in a short period^2^. Thus, understanding the mechanism governing myoblast properties of domestic animals will contribute to the improvement of rearing, feeds, and meat production.

In our study, RNA-seq revealed comprehensive gene expression patterns in WL and UKC myogenic cells during differentiation. We identified the 336 DEGs as the genes differentially expressed between WL and UKC myogenic cells throughout differentiation, suggesting that these genes possibly determine the cellular characteristics of the myogenic cells in each breed. GO analyses showed that the 336 DEGs included a variety of proteins on the cell surface, which can be used as potential markers to select the myoblasts having robust proliferative and differentiative abilities as observed in broilers. Myoblasts are derived from satellite cells existing between the plasma membrane of myofibers and the basal lamina. Recent studies have reported that the microenvironment or niche, such as the extracellular matrix and secreted growth factors, are essential for the maintenance of satellite cells^14^. A latest report indicated that collagen V expressed by murine satellite cells contributes to retain quiescence autonomously^19^. Remarkably, the 336 DEGs contained multiple collagen genes (*COL2A1*, *COL6A3*, *COL9A2*, *COL11A1*, *COL14A1*, *COL16A1*, *COL21A1*, and *LOC426344*). These collagens, differentially expressed between WL and UKC myogenic cells, presumably contribute to create a stem cell niche.

The 336 DEGs also included bone morphogenetic protein (BMP)-binding endothelial cell precursor-derived regulator gene (*BMPER*) and gremlin 2 gene (*GREM2*) that were highly expressed in UKC compared to that in WL myogenic cells. BMP signaling was originally identified as the cascades to generate bones during development^20^, but numerous studies have clarified their functions in various biological processes including musculoskeletal system. In chicken hindlimb buds, BMP signaling is essential to determine the interdigital pattern at late developmental stages^21^. The BMP ligands belonging to transforming growth factor (TGF)-β superfamily are dimeric proteins and bind to heterotetrameric complex of type I and type II BMP receptors, in order to initiate intracellular signaling cascades depending on ligand-receptor combination^22^. Meanwhile, the BMP antagonists extracellularly cleaved and secreted can trap BMP ligands to prevent their binding to BMP receptors and following signal transduction. Spatiotemporal regulation of BMP activities by these antagonists is indispensably required for development of multiple organs^23^. Chicken *BMPER* and *GREM2* genes which are involved in the 336 DEGs encode BMP antagonists. Murine BMPER directly interacts with and antagonizes at least BMP2/4/6 to inhibit endothelial differentiation of embryonic stem cells^24^. Human gremlin 2 antagonizes BMP2/4 to induce myocardial differentiation of pluripotent stem cells by inhibiting SMAD-dependent BMP signaling^25^. These studies demonstrate the key roles of BMP antagonists in non-osteogenic differentiation. Although there has been no information of expression and function about BMPER and gremlin 2 in any myoblasts, BMP2/4/7 are known to inhibit myogenic differentiation in mice^26–28^. Both in layer and broiler chickens, *BMP4* gene expression in hindlimb peaks at HH31-34 (E7-8). Interestingly, temporal raising of incubation temperature during HH23-30 (E4-7) significantly upregulates *BMP4* mRNA levels in both breeds. It results muscle hypertrophy with increasing a number of myofibers in layers, but broilers reduce a number of myofibers and cross-sectional area in gastrocnemius at HH44 (E18)^29^. These data distinctly indicate that elevated BMP signal affects myofiber formation in a breed-dependent manner. From our results, it may be inferred that substantial secretions of BMPER and gremlin 2 from UKC myoblasts would antagonize BMP ligands and enhance muscle formation in broiler chickens.

The 336 DEGs are the gene set that potentially determines the breed-based characteristics of chicken myogenic cells. Further investigation of the 336 DEGs, especially the uncharacterized genes, will lead to identification of genetic markers for selective chicken breeding and will be valuable to understand the mechanism of myogenic differentiation in wide range of animal species.

Furthermore, RNA-seq in our study provided another gene set comprising the 840 DEGs, which are possibly related to myogenic differentiation regardless of the chicken breed. As analyzed using the heatmap, almost all the 840 DEGs indicated parallel expression tendencies but different levels between WL and UKC myogenic cells. This enabled us to classify the 840 DEGs into four subgroups. WG/WD gene levels were altered conspicuously in WL myogenic cells. Transcription levels of UG/UD genes were also altered, especially in UKC myogenic cells. Interactions among the genes were discernibly tighter in UG/UD but not in WG/WD. UG abundantly included cell cycle genes, and UD was rich in muscular components, which corresponds well with the phenotypes of UKC myoblasts.

Generally, in stem cells and their progenies, proliferation and differentiation are inverse processes, which negatively regulate each other^30^. The results of UG/UD do not imply that UKC myoblasts proliferate and differentiate at the same time. UKC myoblasts have a great performance in each program; proliferation in growth condition and differentiation in myogenetic stage. The process of switching from proliferation to differentiation is triggered by the switching/sucrose non-fermenting (SWI/SNF) complex which remodels chromatin and recruits cell type-specific transcription factors. It has been reported that the SWI/SNF complex including BRG1 and PBX1, associates with the myogenic transcription factors MyoD and MEF2 on the murine *Myog* locus^31^. Therefore, the components of the SWI/SNF complex or other chromatin remodelers were expected to be highly expressed in UKC myoblasts. However, we did not find significant differences between WL and UKC myogenic cells regarding this aspect. Numerous studies have revealed that epigenetic modification within the myoblast genome is broadly altered during myogenic differentiation^32^. Thus, genome-wide epigenetic sequencing will present a great deal of insight into the transcriptional differences between WL and UKC myogenic cells.

The DEGs provided the reasonable gene sets illustrating the characteristics of chicken myogenic cells. However, database-based semantic analyses of the DEGs was unable to decipher the importance of the uncharacterized genes. To explore novel factors involved in myogenic properties, we performed PCA independently of the DEGs. PCA successfully defined two major principal components: PC1 demonstrated myogenic differentiation stages, and PC2 governed the differences between WL and UKC. Indeed, the genes with significant factor loadings for PC1 or PC2 displayed some uncharacterized transcripts (designated as “LOC”). Functional investigations of these genes are anticipated to open new avenues for gathering more information about myogenic cells. In addition, a combination of the DEGs and PCA identified the 13 candidate genes representing four peptides or cytokines (*CCK*, *CXCL14*, *MDK*, and *PENK*) and three other genes (*CSRP2*, *MFAP5*, and *UCHL1*), which possibly contribute to the characteristics of chicken myogenic cells.

Cholecystokinin (CCK) is an appetite-regulated hormone which is synthesized predominantly in the gastrointestinal tract. A recent study indicated that rat cardiomyocytes express pro-CCK peptide with a specific N-terminal truncation^33^; however, it has not been reported whether skeletal muscle produce CCK peptides. Administration of CCK-8 to rats increases phosphorylation of AMP-activated protein kinase (AMPK) in skeletal muscle^34^. In murine myoblasts, AMPK phosphorylation attenuates G_1_/S cell cycle transition, inhibits myotube formation, and causes myotube atrophy *via* increasing PGC-1α protein level^35^. In our study, chicken *CCK* gene expression was robustly high in WL myoblasts, especially on day 0 compared to that in UKC myoblasts. Thus, it may be possible that WL myoblast-derived CCK peptides suppress cell proliferation and myogenic differentiation by autocrine signaling.

C-X-C motif chemokine ligand 14 (CXCL14) is a small cytokine expressed in various human tissues including muscle^36^. A recent study indicated that knockdown of murine *Cxcl14* gene induces myogenic differentiation by inhibiting the cell cycle in myoblasts^37^. In this study, RNA-seq revealed that expression levels of chicken *CXCL14* gene are more than ten times higher in UKC than in WL myogenic cells. This may be a partial reason for the active proliferation of UKC myoblasts.

Midkine (MDK) is a heparin-binding growth factor and is expressed with its receptor in myoblasts and myotubes at an early stage of muscle regeneration in rats^38^. *Mdk*^−/−^ mice show delayed muscle regeneration with decreased macrophage infiltration after injury^39^, because MDK serves as a chemotactic protein for migration of immune cells^40^. Chicken *MDK* gene expression in UKC myogenic cells was significantly higher than that in WL myogenic cells during differentiation. Recent studies described acute and chronic inflammatory lesions with infiltration of macrophages and lymphocytes in the breast muscle of broiler chickens^41^. Higher levels of MDK protein produced by UKC myogenic cells may possibly be implicated in the observed muscular inflammation in broilers.

Cysteine- and glycine-rich protein 2 (CSRP2) is a member of the zinc-binding LIM domain protein family. Although CSRP3 promotes myoblast differentiation^42^ and CSRP2 is involved in smooth muscle differentiation^43^, there is no information about CSRP2 in myoblasts. *Csrp2*^−/−^ mice exhibit a slight change in the ultrastructure of cardiomyocytes but show no significant changes in skeletal muscle^44^, which is speculated that CSRP1 and/or CSRP3 may compensate for the lack of CSRP2. Chicken *CSRP2* gene expression tended to be upregulated during differentiation both in WL and UKC myogenic cells. The role of CSRP2 protein in myogenic cells should be elucidated in further studies.

Microfibril associated protein 5 (MFAP5), which is a component of extracellular elastic microfibrils, is downregulated by miR-29. In murine myoblasts, loss of miR-29 promotes transdifferentiation into myofibroblasts with increased *Mfap5* mRNA level^45^. Chicken *MFAP5* gene expression was significantly higher in WL myogenic cells throughout differentiation, which might impair myotube formation, compared to that in UKC.

Ubiquitin C-terminal hydrolase L1 (UCHL1) is expressed not only in nervous systems but also in skeletal muscle. *Uchl1*^−/−^ mice show progressive paralysis and premature death due to the dysfunction of neuromuscular junction^46^. In murine C2C12 myoblast cell line, UCHL1 protein level is downregulated during myogenic differentiation. Knockdown of *Uchl1* gene in C2C12 cells suppresses proliferation and induces myotube formation^47^. While in our study, chicken *UCHL1* transcript levels exhibited more than 10-fold increase during differentiation, both in the WL and UKC myogenic cells. These data indicate that transcriptional regulation of chicken *UCHL1* gene may be different from that in mammals.

In this study, we experimentally focused on *PENK* gene expression in chicken myogenic cells. It has been reported that the rat L6 myoblast cell line consistently expresses *Penk* mRNA^18^. Rat *Penk* gene expression is also detected in skeletal muscle during embryonic and early postnatal development but disappears in adult muscle^48,49^. Herein, we confirmed that expression levels of the avian and mammalian pro-enkephalin genes commonly decline according to myogenic differentiation in primary-cultured embryonic chicken, juvenile murine, and adult human myoblasts. Although their expression patterns throughout life are needed to be further examined in each animal, it seems to be common phenomena that myoblasts produce pro-enkephalin peptide. Two enkephalin derivatives, MENK and LENK, typically serve as opioid peptides *via* classical opioid receptors^15,16^. However, transcriptions of opioid receptor μ (*OPRM1*), δ (*OPRD1*), κ (*OPRK1*), and opioid-related nociceptin receptor 1 (*OPRL1*) were not detected both in WL and UKC myoblasts by RNA-seq (the numbers of the reads <10). On the other hand, an opioid growth factor receptor (*OGFR*) and its homolog (*OGFRL1*) were continuously expressed during the differentiation of chicken myogenic cells. Opioid peptides are also known to act as growth regulators through opioid growth factor receptors in various cellular processes such as development, cancer growth, and angiogenesis^50^. An initial research showed that MENK and LENK peptides inhibit the growth of murine B16-BL6 melanoma to similar extents^51^. Subsequent studies indicated that MENK is a negative growth regulator of murine neuroblastoma^52^ and neural cells^53^. These findings suggest the possibility that the myoblast-derived enkephalins serve as growth factors. Our study revealed that both MENK and LENK peptides suppress proliferation of chicken myoblasts without affecting myogenic differentiation, thereby demonstrating that these enkephalins are novel negative regulators of myoblast growth. In addition to endogenous opioid peptides such as enkephalins, numerous food-derived opioid peptides have been reported^17^. Our results indicate that the opioid peptides present in feeds may possibly influence muscle development of chickens, and the results of murine and human myoblasts expressing enkephalin suggests that it may be a common issue among domestic animals. However, the precise molecular mechanism of the effects of opioids on myoblasts is still unclear. Actions of various opioid peptides and other opioid molecules like alkaloids on myoblasts of widespread species should further be studied.

Using RNA-seq and PCA, we successfully extracted candidate genes that are probably associated with the characteristics of chicken myogenic cells. One of the candidate products, enkephalin, was experimentally proved to suppress the growth of chicken myoblasts. It prompts us to examine the myogenic roles of the other candidate genes. Identification of unknown factors and their functions in myoblasts will contribute to precisely understand the mechanisms of muscle formation and ultimately lead to better meat production.

In this study, we utilized embryonic chicken myoblasts because myofiber formation after hatching can be affected by the given diet, for example^54^. Eliminating extrinsic and environmental factors as possible was critically important for strict comparison between layer and broiler myoblasts. Although body weights of layers and broilers at hatching are significantly different^2,3^, expression of myogenic genes such as myostatin is quite equal in both breeds throughout embryonic development^2,55^. Thus, embryonic myoblasts are considered to be valuable to compare their characteristics. On the other hand, a recent study reported that gene expression patterns of chicken muscle tissue alter even in a short developmental period^56^. Investigation of gene expression profiles in the isolated myoblasts at various developmental stages will clarify the myoblast characteristics of the breeds more accurately.

## Methods

### Myoblast culture

All experimental procedures were conducted in accordance with the Regulations for Animal Experimentation of Shinshu University, and the animal experimentation protocol was approved by the Committee for Animal Experiments of Shinshu University.

Myoblasts from hindlimb muscles of WL or UKC chicken embryos at HH36 (E10) were isolated, primary-cultured, and induced to differentiate as previously described^57,58^. Conventional WL and UKC chicken eggs were provided by National Federation of Agricultural Cooperative Associations (Tokyo, Japan) and were conventionally cultured at 38.5 °C and 60% humidity with 45° horizontal rotation every 3.75 min in an incubation chamber for 10 days. Ten embryos were served for myoblast isolation in each line to equalize the individual differences and to overcome a small cell number per embryo. Femora of the embryos were amputated at hip and knee joints. After peeling skins and removing thigh bones, femoral muscles were minced in collagenase solution consisting of 2 mg/ml collagenase II (Sigma-Aldrich, MO, USA), DMEM (Nacalai, Osaka, Japan), 10% fetal bovine serum (FBS) (HyClone; GE Healthcare, UT, USA), and a mixed solution of 100 units/ml penicillin and 100 μg/ml streptomycin (PS) (Nacalai). After incubation at 37 °C for 1 h, cell suspension was filtered using 100-μm Cell Strainer (Thermo Fisher Scientific, MA, USA) to eliminate myofibers. The filtered cells were suspended in growth medium (GM) consisting of RPMI1640 (Nacalai), 20% FBS, 1% non-essential amino acids (Wako, Osaka, Japan), 1% chicken embryo extract (US Biological, MA, USA), 2 ng/ml recombinant human basic fibroblast growth factor (rh-bFGF) (Wako), and PS. The cell suspension was incubated at 37 °C with 5% CO_2_ for 24 h on non-coated dishes for 24 h to paste adherent cells including adipocytes, fibrocytes, and vascular cells but not myoblasts which do not attach to non-coated dishes. Then, chicken myoblasts in supernatant were cultured on the dishes or plates coated with collagen type I-C (Cellmatrix; Nitta Gelatin, Osaka, Japan). Chicken myoblasts were cultured at 37 °C with 5% CO_2_ throughout the experiments, which is according to previous studies and the report that mRNA levels of myogenic genes in chicken femoral myoblasts at 37 °C were not significantly different from those at 39 °C^59^. Undifferentiated chicken myoblasts were maintained in GM. Myogenic differentiation of myoblasts was induced as follows. The chicken myoblasts maintained in GM were dissociated by treating with 0.25% trypsin with 1 mM EDTA (Wako) for 5 min at 37 °C. Then the myoblasts suspended in GM were seeded on collagen type I-C-coated dishes or plates at appropriate cell numbers (see each experimental method) to equalize confluency among the samples. The myoblasts in GM after 24 h of seeding were defined as day 0. Myogenic differentiation was induced by replacing GM with differentiation medium (DM) consisting of DMEM, 2% FBS, and PS.

Primary-cultured murine myoblasts from skeletal muscle of 4-week-old C57BL/6 J mice were isolated, maintained, and induced to differentiate as previously described^56^. 5.0 × 10^5^ murine myoblasts were cultured on the collagen type I-C-coated 60-mm dishes and maintained in GM comprising Ham’s F10 medium (Thermo Fisher Scientific), 20% FBS, 2 ng/ml rh-bFGF, and PS. Murine myoblasts were induced to differentiate in DM consisting of DMEM, 5% FBS, and PS.

Primary-cultured adult human myoblasts were purchased (CC-2580; Lonza, MD, USA) and cultured according to the manufacturer’s instruction. In brief, 2.0 × 10^5^ human myoblasts were seeded on collagen type I-C-coated 60-mm dishes and maintained in Skeletal Muscle Growth Media-2 (CC-3245; Lonza) including 30 μg/ml gentamicin and 15 ng/ml amphotericin B. Human myoblasts were induced to differentiate in DM consisting of DMEM, 2% horse serum (HyClone; GE Healthcare), and PS.

### Cell counting

2.5 × 10^4^ chicken myoblasts/well were seeded on collagen type I-C-coated 12-well plates. The myoblasts were continuously cultured in GM until their numbers counted. For counting, the cells were completely dissociated by treating with 0.25% trypsin with 1 mM EDTA (Wako) for 5 min at 37 °C. The numbers of myoblasts were counted using a hemocytometer. These dissociated cells were not seeded again.

### EdU staining

1.0 × 10^5^ chicken myoblasts were seeded on collagen type I-C-coated 30-mm dishes. After 24 h, EdU (5-ethynyl-2′-deoxyuridine) was added at the final concentration of 10 μM, and the cells were cultured for 3 h. EdU staining was performed using Click-iT EdU Imaging Kit (Thermo Fisher Scientific) according to the manufacturer’s instruction. Cell nuclei were visualized by DAPI staining. The ratio of EdU^+^ cells were defined as the number of EdU^+^ nuclei divided by the total number of nuclei (155.3 nuclei/field in average × four images in each experiment) using ImageJ software (National Institutes of Health, USA).

### MHC staining

1.0 × 10^5^ chicken myoblasts were seeded on collagen type I-C-coated 30-mm dishes, and subsequently myogenic differentiation was induced for 2 days. The cells were fixed with 2% paraformaldehyde for 5 min at room temperature (rt), and permeabilized by 0.2% Triton X-100 (Nacalai) for 5 min at rt. After blocking with 1% bovine serum albumin for 30 min at rt, the cells were treated with 0.5 μg/ml mouse monoclonal anti-MHC antibody MF20 (R&D Systems, MN, USA) overnight at 4 °C. Finally, the cells were treated with 1 μg/ml Alexa Fluor 488-conjugated donkey anti-mouse IgG antibody (Jackson ImmunoResearch, PA, USA) for 1 h at rt to detect MHC. Cell nuclei were visualized by DAPI staining. Phase-contrast and fluorescent images were taken and layered using EVOS FL Auto microscope (AMAFD1000; Thermo Fisher Scientific). The rate of MHC^+^ cells was defined as the number of nuclei in the MHC^+^ cells divided by the total number of nuclei (171.7 nuclei/field in average × four images in each experiment), and the fusion index was defined as the number of nuclei in multinuclear MHC^+^ myotubes divided by the total number of nuclei using ImageJ software.

### RNA-seq

For RNA sampling, 2.0 × 10^5^ chicken myoblasts were seeded on the collagen type I-C-coated 60-mm dishes, and myogenic differentiation was induced as described above. Total RNA of the myogenic cells was isolated using NucleoSpin RNA Plus (Macherey-Nagel, Düren, Germany) on days 0, 1, and 2. RNA qualities were checked using Agilent 2100 Bioanalyzer and Agilent RNA 6000 nano kit (Agilent Technologies, Waldbronn, Germany). RNA integrity number (RIN) values were >8.0 in all samples (Supplementary Fig. S1). 1 μg of total RNA was subjected to prepare libraries for RNA-seq using Illumina TruSeq RNA Library Prep Kit v2 (Illumina, CA, USA). Library qualities were confirmed using Agilent 2100 Bioanalyzer and Agilent DNA 1000 Reagents (Agilent Technologies) (Supplementary Fig. S2). 4 nM of the libraries were denatured, and subsequently subjected to RNA-seq. RNA-seq was performed using Illumina HiSeq. 2500 (Illumina) with conditions kept at 102 cycles and single reads. FASTQ read data was generated using bcl2fastq conversion software 2.18 (Illumina). The quality of the read (Q score) was calculated as the arithmetic mean of its Phred quality scores. After removing low-quality reads (Q score < 36) and trimming the adaptor sequences from raw reads, the processed data were mapped to a reference genome (Gallus_gallus-5.0 Assembly; GCA_000002315.4). Mapping efficiencies are shown in Supplementary Table S1. The mapped reads were subjected to transcript annotation, and expression levels were calculated using the RPKM method by CLC Genomics Workbench 9.5.2 (Qiagen, Hilden, Germany). FDR was employed to correct the *p* values.

### Volcano plotting

Volcano plotting is used to show a differential gene expression pattern between a pair of the samples. The pair was described as X vs Y (Fig. 2D,E), in which X and Y represents the sample name. The difference in gene expression was plotted as the logarithm of fold-change to base 2 (X-axis) and the minus value of common logarithm of *p* value (Y-axis).

### GO analysis

The DEGs were subjected to GO analysis using DAVID Bioinformatics Resources 6.8 (https://david.ncifcrf.gov/)^60^. The GO terms in biological processes having *p* value <0.05 were defined as significantly enriched gene clusters.

### Heatmap generation

Heatmap of the expression levels of the 840 DEGs was generated by Heatmapper (http://www.heatmapper.ca/)^61^ with the following settings: Clustering method, Average linkage; Distance measurement method, Pearson. Each row represents a gene, and each column represents an average of triplicate samples. The green and red gradients indicate an increase and decrease in gene expression, respectively.

### STRING analysis

Functional and physiological interactions of the genes were visualized using STRING version 10.5 (https://string-db.org/)^62^.

### PCA

The RPKM values of RNA-seq (with the numbers of reads >0) were used for PCA. In total 13,815 genes were used as the number of dimension for the 18 sample vectors (WL and UKC samples on days 0, 1, and 2 in triplicates). Each vector contained the RPKM values as the elements. Variance-covariance matrices (13,815 × 13,815 dimension) were calculated from the 18 vectors. The matrices were diagonalized, and the eigen values and vectors were obtained. The projection on the eigen vector of the largest eigen value corresponded to the first component of PCA (PC1). The second component of PCA (PC2) was the projection on the eigen vector of the second largest eigen value. These calculations were done using Python scripts.

### qPCR

The total RNA of chicken myoblasts used for RNA-seq was utilized for qPCR. Total RNA from murine and human myoblasts was isolated using TRIzol Reagent (Thermo Fisher Scientific). RNA was reverse transcribed using ReverTra Ace qPCR RT Master Mix (TOYOBO, Osaka, Japan). qPCR was performed using GoTaq qPCR Master Mix (Promega, WI, USA) with StepOne Real-Time PCR System (Thermo Fisher Scientific). The amount of each transcript was normalized to that of chicken 16 S ribosomal RNA (*16 S*), murine 18 S ribosomal RNA (*Rn18S*), or human tyrosine 3-monooxygenase/tryptophan 5-monooxygenase activation protein zeta gene (*YWHAZ*). The results are presented as fold-change. Primer sequences are described in Supplementary Table S3.

### Chemicals

MENK (Tyr-Gly-Gly-Phe-Met) (Peptide Institute, Osaka, Japan) and LENK (Tyr-Gly-Gly-Phe-Leu) (Wako) were dissolved in sterile phosphate-buffered saline (PBS). In enkephalin treatment experiments, equal volumes of sterile PBS instead of the test samples served as negative control.

### Statistical analysis

The results were presented as mean ± standard error. Statistical differences between two groups were evaluated by unpaired two-tailed Student’s *t* test, and statistical differences among more than three groups were evaluated by Williams’ test or Scheffe’s *F* test following one-way analysis of variance (ANOVA) using KaleidaGraph software (Synergy Software, PA, USA). Statistical significance was set at *p* value <0.05.

## Supplementary information

Supplementary Information

Supplementary Data 1

Supplementary Data 2

## Data Availability

FASTQ read data of RNA-seq in this study are deposited in DDBJ Sequence Read Archive (DRA; Research Organization of Information and Systems, National Institute of Genetics, Mishima, Japan) with the accession number: DRA007964.
