# Supplementary material for: Distinct cell proliferation, myogenic differentiation, and gene expression in skeletal muscle myoblasts of layer and broiler chickens
Source: Sci Rep. 2019 Nov 11;9:16527. doi: 10.1038/s41598-019-52946-4 (PMC6848216; doi:10.1038/s41598-019-52946-4)

Supplementary Information

Distinct cell proliferation, myogenic differentiation, and gene expression in skeletal muscle myoblasts of layer and broiler chickens

Yuma Nihashi[1], Koji Umezawa[2,3], Sayaka Shinji[1], Yu Hamaguchi[4], Hisato Kobayashi[4†], Tomohiro Kono[5], Tamao Ono[1,2], Hiroshi Kagami[2] & Tomohide Takaya[1,2,3*]

[1]Department of Agriculture, Graduate School of Science and Technology, Shinshu University, 8304 Minami-minowa, Kami-ina, Nagano 399-4598, Japan.
[2]Department of Agricultural and Life Science, Faculty of Agriculture, Shinshu University, 8304 Minami-minowa, Kami-ina, Nagano 399-4598, Japan.
[3]Department of Interdisciplinary Genome Sciences and Cell Metabolism, Institute for Biomedical Sciences, Shinshu University, 8304 Minami-minowa, Kami-ina, Nagano 399-4598, Japan.
[4]NODAI Genome Research Center, Tokyo University of Agriculture, 1-1-1 Sakuragaoka, Setagaya-ku, Tokyo 156-8502, Japan.
[5]Department of Bioscience, Tokyo University of Agriculture, 1-1-1 Sakuragaoka, Setagaya-ku, Tokyo 156-8502, Japan.

† Present address: Department of Embryology, Nara Medical University, 840 Shijo-cho, Kashihara, Nara, 634-8521 Japan.

*Correspondence and requests should be addressed to T.T. (email: ttakaya@shinshu-u.ac.jp)

Supplementary References

1. Nihashi, Y., Ono, T., Kagami, H. & Takaya, T. Toll-like receptor ligand-dependent inflammatory responses in chick skeletal muscle myoblasts. *Dev. Comp. Immunol.* **91**, 115-122 (2019).
2. Hirai, H. *et al.* MyoD regulates apoptosis of myoblasts through microRNA-mediated down-regulation of Pax3. *J. Cell Biol.* **191**, 347-365 (2010).
3. Baptista, F. I., Pinto, M. J., Elvas, F., Almeida, R. D. & Ambrosio, A. F. Diabetes alters KIF1A and KIF5B motor proteins in the hippocampus. *PLoS One* **8**, e65515 (2013).

Table S1. The numbers of the reads obtained by RNA-seq.

Sample	No. of input reads	No. of mapped reads	% mapped
UKC P04 day 0	21,812,633	21,148,264	97.0%
UKC P04 day 1	22,489,004	21,835,912	97.1%
UKC P04 day 2	24,764,717	24,103,965	97.3%
UKC P05 day 0	21,189,864	20,571,250	97.1%
UKC P05 day 1	22,606,963	21,975,686	97.2%
UKC P05 day 2	25,375,970	24,697,715	97.3%
UKC P07 day 0	23,073,691	22,380,689	97.0%
UKC P07 day 1	22,275,274	21,667,048	97.3%
UKC P07 day 2	24,544,406	23,885,619	97.3%
WL P08 day 0	24,636,499	23,929,133	97.1%
WL P08 day 1	22,279,784	21,709,664	97.4%
WL P08 day 2	21,924,068	21,378,021	97.5%
WL P09 day 0	23,197,326	22,560,790	97.3%
WL P09 day 1	22,870,723	22,246,047	97.3%
WL P09 day 2	24,432,387	23,831,100	97.5%
WL P10 day 0	24,958,361	24,198,953	97.0%
WL P10 day 1	22,996,430	22,362,891	97.2%
WL P10 day 2	23,271,079	22,694,664	97.5%

Table S2. Gene ontologies of UG and UD subgroups.

**Group**	**Ontology**	***p***
UG	Cell cycle process	1.2.E-04
	Cell cycle	7.7.E-04
	Chromosome organization	5.9.E-03
	Mitotic nuclear division	6.4.E-03
	Organelle organization	7.2.E-03
	Mitotic cell cycle	1.3.E-02
	Cell division	1.3.E-02
	Single-organism organelle organization	1.4.E-02
	DNA conformation change	1.6.E-02
	DNA integrity checkpoint	2.0.E-02
	Cellular response to DNA damage stimulus	4.7.E-02
UD	Muscle contraction	1.2.E-04
	Regulation of muscle contraction	1.6.E-03
	Regulation of system process	1.6.E-03
	Striated muscle contraction	2.4.E-03
	Regulation of multicellular organismal process	1.4.E-02

Table S3. Primer sequences for qPCR.

Gene	Sequence (5'-3')	Reference
16S (chicken)	ACCTATTTGACTCCCTCAACCA<br>AAGTTTACGCCGTAGGAGGATAGGTT	Previously reported[1]
PENK (chicken)	GCCCAGAACTGGAAGATGAA<br>TCTCTGGGACCTCTTTGGAA	This study
Rn18s (mouse)	CGCACGGCCGGTACAGTGAAACTG<br>CACCCGTGGTCACCATGGTAGGCA	Previously reported[2]
Penk (mouse)	CAGGCGACATCAATTTCCTG<br>TCATCCTGTTTGCTGCTGTC	This study
YWHAZ (human)	CAAGCATACCAAGAAGCATTTGA<br>GGGCCAGACCCAGTCTGA	Previously reported[3]
PENK (human)	CATCCTCCAGTGGGAAACTG<br>CCAAAAAGAGCACAGAACCTG	This study

Supplementary Figure legends

Figure S1. Qualities of the RNA samples used for RNA-seq. The RIN values measured by Agilent 2100 Bioanalyzer are indicated.

Figure S2. Qualities of the sequence libraries. All libraries had a single peak and no adaptor dimers.

Figure S1

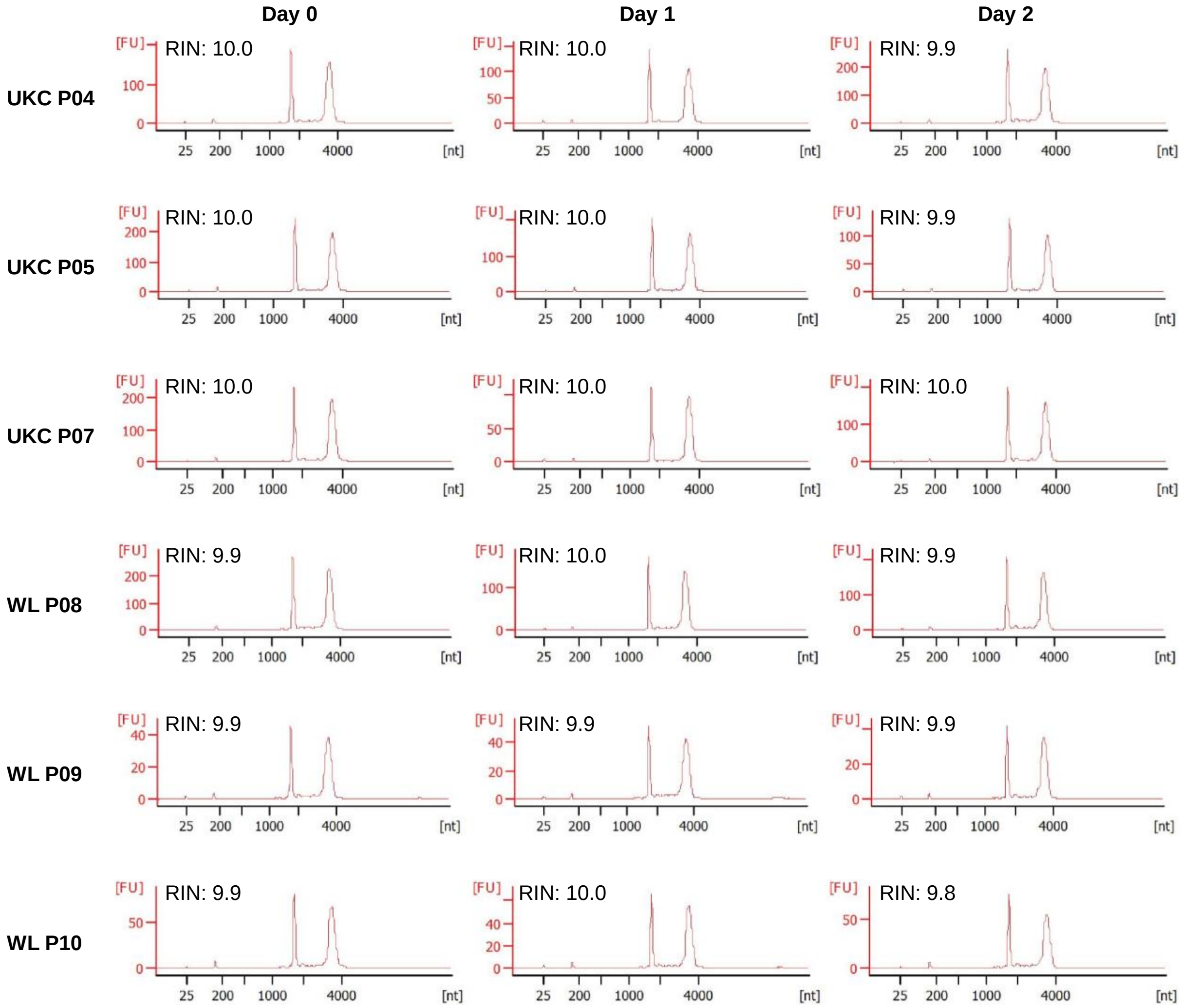
Day 0
Day 1
Day 2
UKC P04
RIN: 10.0
RIN: 10.0
RIN: 9.9
UKC P05
RIN: 10.0
RIN: 10.0
RIN: 9.9
UKC P07
RIN: 10.0
RIN: 10.0
RIN: 10.0
WL P08
RIN: 9.9
RIN: 10.0
RIN: 9.9
WL P09
RIN: 9.9
RIN: 9.9
RIN: 9.9
WL P10
RIN: 9.9
RIN: 10.0
RIN: 9.8
[FU]
[nt]
25
200
1000
4000

Figure S2

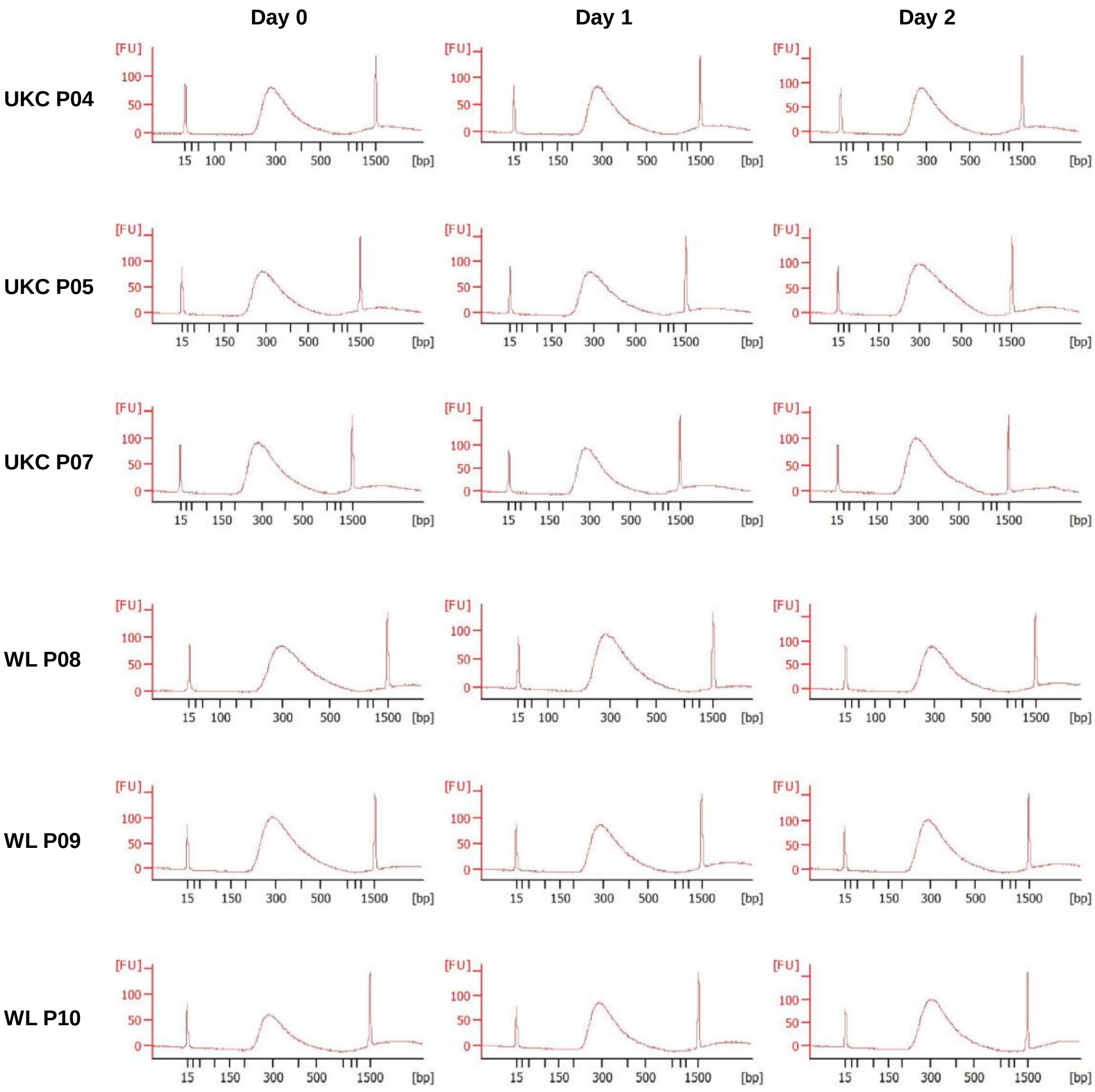

Supplement: Supplementary file 1 — Supplementary Information [file 41598_2019_52946_MOESM1_ESM.pdf]
